# The Contribution of the Locus Coeruleus–Noradrenaline System Degeneration during the Progression of Alzheimer’s Disease

**DOI:** 10.3390/biology11121822

**Published:** 2022-12-14

**Authors:** Dilek Mercan, Michael Thomas Heneka

**Affiliations:** 1 German Center for Neurodegenerative Diseases (DZNE), 53127 Bonn, Germany; 2 Luxembourg Centre for Systems Biomedicine, University of Luxembourg, 4365 Esch-sur-Alzette, Luxembourg; 3 Division of Infectious Diseases and Immunology, University of Massachusetts Medical School, Worcester, MA 01605, USA

**Keywords:** Alzheimer’s disease, locus coeruleus, noradrenaline, norepinephrine, cognition, neurodegeneration, neuroinflammation

## Abstract

**Simple Summary:**

Locus coeruleus degeneration occurs early in the course of Alzheimer’s disease and likely contributes to pathogenic progression. Involved mechanisms may therefore be targeted for future therapies.

**Abstract:**

Alzheimer’s disease (AD), which is characterized by extracellular accumulation of amyloid-beta peptide and intracellular aggregation of hyperphosphorylated tau, is the most common form of dementia. Memory loss, cognitive decline and disorientation are the ultimate consequences of neuronal death, synapse loss and neuroinflammation in AD. In general, there are many brain regions affected but neuronal loss in the locus coeruleus (LC) is one of the earliest indicators of neurodegeneration in AD. Since the LC is the main source of noradrenaline (NA) in the brain, degeneration of the LC in AD leads to decreased NA levels, causing increased neuroinflammation, enhanced amyloid and tau burden, decreased phagocytosis and impairment in cognition and long-term synaptic plasticity. In this review, we summarized current findings on the locus coeruleus–noradrenaline system and consequences of its dysfunction which is now recognized as an important contributor to AD progression.

## 1. Alzheimer’s Disease

Alzheimer’s disease (AD) is the most common type of dementia and it is characterized by progressive loss of memory and other cognitive functions. Globally, more than 40 million people are affected by AD and it is predicted that this number will be tripled by 2050 [1]. Epidemiological studies indicated that AD prevalence is 10–30% in the age group over 65 with an incidence of 1–3% [2]. While a large number of the AD patients (more than 95%) who are at 80–90 years of age present the sporadic form of the disease, a small proportion (less than 1%) of AD patients suffers from autosomal dominant inherited AD with an age of onset approximately 3–4 decades before. AD belongs to the group of neurodegenerative proteinopathies and is characterized by the extracellular accumulation of amyloid-β (Aβ), forming amyloid deposits within the brain parenchyma, and abnormally phosphorylated tau protein, leading to an intraneuronal formation of neurofibrillary tangles (NFTs) [3].

**Figure 1:**
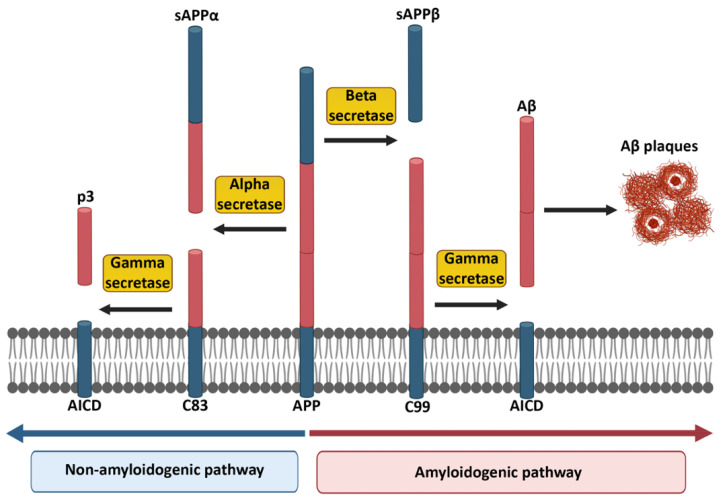
Non-amyloidogenic and amyloidogenic pathways in the cleavage of APP. The red part of APP indicates the potentially harmful Aβ portion, which appears at the end of the amyloidogenic pathway. Created partially with BioRender.com (accessed on 28 June 2021).

Aβ, which is the main constituent of the extracellular amyloid plaques, is derived from amyloid precursor protein (APP) as a result of proteolytic cleavage. APP, which is a transmembrane protein, is expressed in the brain by several cell types, including neurons, astrocytes and microglia, but also endothelial cells lining brain vessels. APP is also expressed in peripheral organs including the adrenal gland, heart, pancreas, liver, kidney, spleen [4,5]. Nevertheless, the majority of studies has focused on the brain where APP and its cleavage products play crucial roles in synaptic function, neurogenesis, and plasticity [6,7]. APP is processed in two different ways. In the nonamyloidogenic pathway, APP is cleaved by alpha secretase (α-secretase), which results in the cleavage products sAPPα and C83 (also known as α-secretase C-terminal fragment “α-CTF”). While sAPPα is released into the extracellular compartment, C83 stays in the membrane. This remaining part is subsequently recognized and cleaved into p3 and APP intracellular domain (AICD) by gamma secretase (γ-secretase). Due to the fact that this cleavage is performed within the Aβ protein sequence, the nonamyloidogenic pathway does not lead to Aβ formation (Figure 1). It is important to mention that sAPPα has important physiological properties, including neurotrophic support, neuroprotection, that enhance long-term potentiation (LTP) and memory, modulation of spine density, and promote neurite and axonal outgrowth [8]. The toxic Aβ is generated in the amyloidogenic pathway, in which APP is sequentially cleaved by beta secretase (β-secretase, also known as beta-site APP cleaving enzyme, BACE) which results in the cleavage products sAPPβ and C99. Subsequent γ-secretase cleavage of C99 leads to the formation of AICDs and Aβ monomers (Figure 1) [9,10]. It is noteworthy to state that imprecise γ-secretase cleavage of C99 fragment leads to the formation of different Aβ peptides with different amino acid lengths, such as Aβ30, Aβ34, Aβ38, Aβ39, Aβ40, Aβ42, Aβ45, Aβ48, Aβ51, of which the longest Aβ peptide is Aβ51, whereas the shortest is Aβ30 [11].

APP cleavage products play important roles in the brain. For example, APPα regulates calcium (Ca^2+^) homeostasis under hypoxic conditions [12] and AICD is involved in lipid biosynthesis [13]. The accumulation of pathogenic Aβ peptides represents a hallmark of AD and this pathology develops as a consequence of failure to clear Aβ, in particular the Aβ42 isoform. Aβ is an important factor in the development of AD and found in 99% of the AD patients [14]. It is important to mention that Aβ42 is the most aggregation-prone and the most toxic species among different Aβ peptides. In addition, while Aβ42 is dominant in the brain, Aβ40 is mostly found in the periphery [15]. Over the years, there have been numerous studies to investigate the genetic factors contributing to AD progression, such as mutations in APP and presenilins [16,17,18].

**Figure 2:**
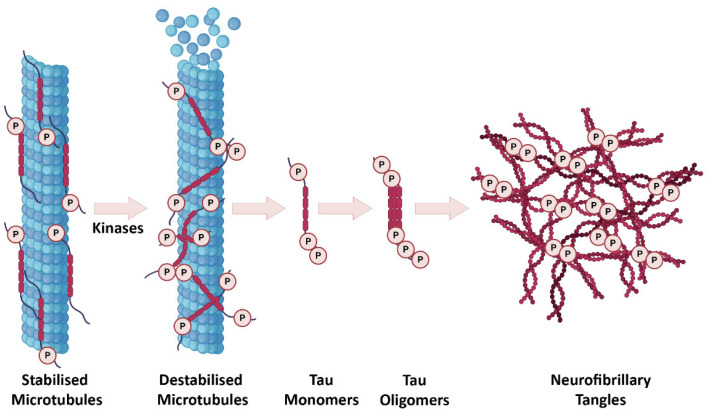
The progression of tau pathology in AD. In a healthy brain, tau is involved in regulation of microtubule stabilization. After tau hyperphosphorylation, microtubules are destabilized and start to disassemble. Tau monomers and oligomers subsequently occur intraneuronally and eventually formation of NFTs takes place. Created partially with BioRender.com (accessed on 28 June 2021).

In a healthy brain, tau interacts with tubulin to promote microtubule stabilization and assembly [19]. During AD pathogenesis, tau is abnormally phosphorylated leading to the formation of pathological soluble tau oligomers, which form subsequent insoluble globular tau oligomers within the neuron. As a result of continuous intraneuronal tau aggregation, from the point of globular tau, oligomers first paired helical filaments and finally NFTs are formed (Figure 2) [20,21,22]. Tau monomers can also be found in the extracellular space, secreted in an active process, and can be taken up by neighboring neurons [21,23,24,25]. In this way, spreading of tau pathology into neighboring neurons might occur. Tau is mainly an axonal protein but its pathological form accumulates in the soma where it is usually less abundant. Hyperphosphorylated soluble tau impairs the axonal transport [26] and prevents microtubule assembly [27]. One study also showed that the accumulation of hyperphosphorylated tau is observed within dendritic spines, which gives rise to synaptic dysfunction through the impairment of glutamate receptor trafficking [28]. 

## 2. The Role of Microglia in Alzheimer’s Disease

Neuroinflammation plays a pivotal role in the progression of AD. A series of recent studies has indicated that AD displays a sustained inflammatory response [29,30,31,32,33,34,35]. Chronic inflammation in AD takes place as a result of imbalanced anti-inflammatory and pro-inflammatory signaling and is linked to activated microglia. The latter, which derive from precursors in the embryonic yolk sac during primitive hematopoiesis, are the resident innate immune cells in the brain [36,37,38]. 

In light of various studies, it has been reported that microglia harbor a great adaptation property and they can quickly alter their functions according to different conditions [39,40]. Under healthy physiological conditions, when no immune activating signal is present, microglia possess a ramified phenotype with large numbers of highly arborized long processes and a small soma size [41]. This type of microglia is called quiescent/resting/surveying or, more recently, homeostatic microglia. These microglia continuously scan the brain parenchyma and interact with nearby blood vessels, oligodendrocytes, astrocytes and neurons. This is very important because, this way, microglia can provide a rapid response to any alteration in the microenvironment and preserve the brain homeostasis, which includes clearing misfolded proteins, cellular debris and dead/dying cells using their enhanced phagocytic capability [42,43,44,45,46,47,48]. In case microglia detect a threat, they initiate defense mechanisms and become activated, which gives rise to several morphological changes including increased motility, retraction of their ramified processes and increased soma size [46,49,50,51]. 

**Figure 3:**
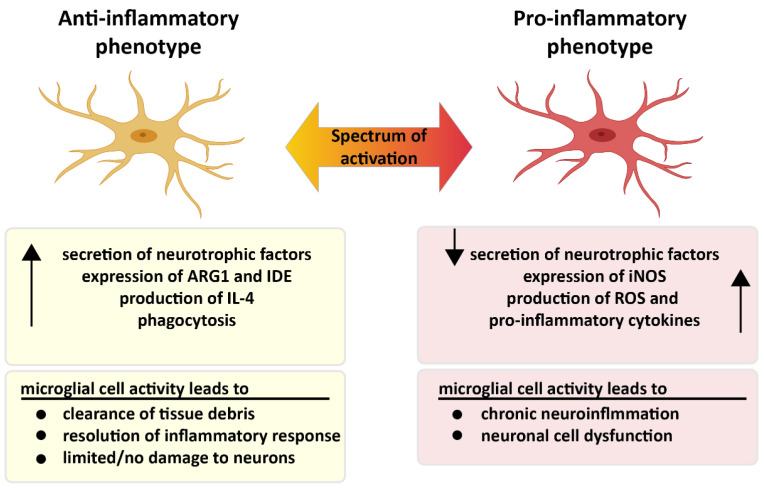
Two different faces of microglia. Pro- and anti-inflammatory microglia display different characteristics in the presence of microglial activation. While microglia are neuroprotective in the anti-inflammatory state, microglia in the pro-inflammatory condition trigger chronic inflammation, which leads to neuronal dysfunction. Created partially with BioRender.com (accessed on 28 June 2021).

Microglia exhibit many different phenotypes, where pro- and anti-inflammatory phenotypes of microglia represent two extreme states of a broad spectrum (Figure 3). Activated microglia mainly produce pro-inflammatory cytokines and chemokines including but not restricted to interleukin (IL)-1β, IL-6, IL-12, IL-18, CCL2, TNF-α, interferon (IFN)-γ and free radicals such as nitric oxide (NO) and superoxide anions [52,53]. Moreover, major histocompatibility complex-II (MHC-II), integrins (CD11b, CD11c), costimulatory molecules (CD36, CD45, CD47) can also be expressed by activated microglia [52]. Although pro-inflammatory responses play an important role in neurodegenerative diseases or protective mechanisms against brain injury, pathogens and tumors, they can harm healthy cells, such as neurons and other glial cells in particular, when present for prolonged periods of time [54]. As has been previously reported in the literature, sustained release of pro-inflammatory molecules leads to inhibition of neurogenesis [55], defects in axonal transport and cell death [56]. A large number of previous studies have shown that: microglia produce anti-inflammatory cytokines such as IL-4, IL-10; express arginase 1 (ARG1) and insulin-degrading enzyme (IDE); secrete several growth factors including transforming growth factor-β (TGF-β), insulin-like growth factor-1 (IGF-1), fibroblast growth factor (FGF), colony stimulating factor-1; and release neurotrophic growth factors (nerve-derived growth factor [NGF], brain-derived neurotrophic factor [BDNF], neurotrophins, glial cell–derived neurotrophic factor [GDNF]). In this activated state, the phagocytic activity of microglia is enhanced [52,57]. Recently, it has been suggested that perturbations in switching from pro- to anti-inflammatory microglial phenotypes might cause the chronic inflammation, which is the situation mostly seen in neurodegenerative diseases [58].

Aβ is an important factor causing microglial activation in AD progression. As a result of the activation, microglia initially migrate to the plaques and initiate Aβ removal by phagocytosis and degradation [49,59,60]. However, after prolonged duration of activation, they lose their ability to clear Aβ deposits [61,62] as a consequence of continued inflammatory activation [63]. Constant microglia activation gives rise to chronic neuroinflammation and worsening of AD pathology with sustained change of the microglial pool. For instance, in the APP/PS1 mouse model, a new type of microglia called “dark microglia“ was identified at the vicinity of Aβ plaques, which displayed an extremely activated phenotype with elevated levels of CD11b and TREM2 (triggering receptor expressed on myeloid cells-2) expressions, when they are close to Aβ plaques [64]. This is a clear hint that various subpopulations of microglial phenotypes exist with their different functions in the AD brain [64,65]. Several years ago, a novel subtype of microglia, called disease-associated microglia (DAM), was described in 5XFAD transgenic mice expressing five human familial AD gene mutations [66]. In the subsequent years, the DAM phenotype was also reported in further murine AD models as well as in human AD brains [67]. In the study, a DAM population was identified within proximity to Aβ plaques using sub-tissue-focused single-cell RNA-seq and smFISH techniques [66]. A detailed analysis of gene expression profiles of DAM showed an elevation of lipid metabolism pathways and phagocytic-related genes, which may be associated with amyloid clearance during AD progression. It was pointed out that activation of DAM occurs in two steps. The first step, which is Trem2 independent, involves activating a number of genes including Trem2-signaling adaptor Tyrobp, Apoe, and B2m, along with downregulating microglia homeostatic genes (e.g., Cx3cr1 and P2ry12/P2ry13). The second step, which is Trem2-dependent, involves the induction of lipid metabolism and phagocytic pathways (e.g., Lpl, Cst7, and CD9) [66]. It is worth noting, however, that a recently published perspective paper on microglia states is somewhat skeptic about the naming of microglia subtypes [68]. For instance, it was argued that microglial acronyms, such as DAM, might present a conceptual weakness, since they suggest stable states or phenotypes of microglia and do not reflect the dynamic changes these cells likely undergo during chronic neuroinflammation and neurodegeneration. In addition, it is also known that they are very heterogeneous and plastic. Another point of discussion regarding the use of this DAM as a characterizing term is whether those microglia are associated with specific disease entities or distinct pathological challenges [68].

In AD, activated microglia secrete cytokines [69]. This phenomenon is mimicked in related murine models; thus, in TgAPPsw and PS1/APPsw mice, elevated levels of Aβ are correlated with increased concentrations of pro-inflammatory cytokines, including but not restricted to IL-1α, IL-1β IL-6, TNF-α and granulocyte-macrophage colony-stimulating factor [70]. Aβ per se is known to increase production of cytokines through ligation of pattern recognition receptors on the microglia surface. Treatment of microglia with Aβ42 enhanced the production of pro-IL-1β, IL-6, TNF-α, monocyte chemoattractant protein-1 (MCP-1), macrophage inflammatory peptide-1α (MIP-1α), IL-8, and macrophage colony-stimulating factor, which are all pro-inflammatory immune mediators [71]. It is important to note that inflammatory cytokines may compromise neuronal function, for instance, by suppressing synaptic plasticity [69,72] and may even cause neuronal death [73], in particular when co-occurring together with excitotoxic stimuli [74]. 

## 3. The Locus Coeruleus

**Figure 4:**
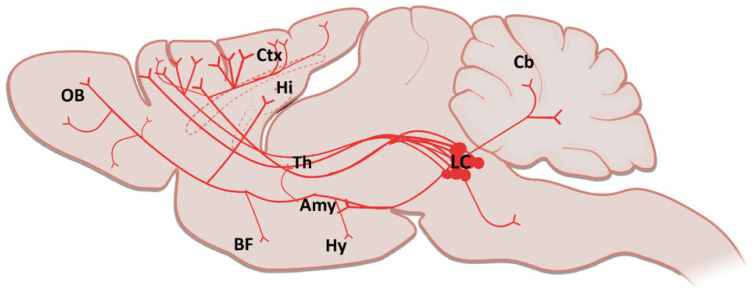
A sagittal schematic illustration showing the LC projections in the brain (Amy: Amygdala; BF: Basal Forebrain; Cb: Cerebellum; Ctx: Cortex; Hi: Hippocampus; Hy: Hypothalamus; LC: Locus Coeruleus; Th: Thalamus, OB: Olfactory Bulb). Created partially with BioRender.com (accessed on 28 June 2021).

The Locus Coeruleus (LC) is a well described brain stem nucleus located near the tectum of the fourth ventricle [75]. Although the LC contains only a small number of neurons, about 1500 in a rat and 10.000–15.000 in a human [76], it sends widespread projections to various regions in the central nervous system (CNS) including the thalamus [77,78], hypothalamus [79,80,81], spinal cord [82], cerebellum [83], orbitofrontal (OFC), medial prefrontal (mPFC), anterior cingulate (ACC) cortices, primary motor cortex (M1) and hippocampus [84,85,86,87] (Figure 4). This small brainstem structure is involved in several important brain functions including sleep architecture, arousal, vigilance, selective attention, learning, memory consolidation, modulation of synaptic plasticity, stress reactivity, behavioral adaptation and regulation of inflammation [88,89,90].

The LC, the major noradrenergic nucleus of the brain, has a heterogeneous internal organization for its projection targets. Cells which project to the hippocampus and septum are mostly localized in the dorsal part of the LC, while the cerebellum receives projections from both the dorsal and ventral side of the LC. In addition to this, the thalamus and hypothalamus receive projections from caudal and rostral areas of the LC, respectively. Although LC neurons are known to exhibit cortical projections, which are scattered throughout the nucleus, recent studies showed that those projections prominently arise from caudal poles of the LC [91,92,93]. Moreover, the LC sends projections throughout the brain in different extents. For example, retrograde tract-tracing experiments in rats showed that 60.8% of retrogradely labeled LC cells projected to mPFC, 35.4% projected to M1 and 3.9% projected to both regions [86]. Furthermore, the LC cells which are sending projections to mPFC have a three times faster discharging capacity than those projecting to M1, which may give rise to three-fold difference in NA concentration between these two regions [86]. Besides, expression levels of dopamine β-hydroxylase (DBH) mRNA were shown to be strikingly similar between the cells projecting to mPFC and M1, which means both regions have an equivalent capacity for NA production from dopamine [86]. These findings are critical to understanding the role of the LC in different brain regions.

The LC regulates the sleep and wake cycle, thereby promoting maintenance of arousal. Studies show that the LC shows almost no activity during the sleep phase [94]. As has been previously reported using mainly in vivo electrophysiological recordings, LC neurons are less active during non–rapid eye movement (NREM) sleep and almost silent during REM (rapid eye movement) sleep phase [95,96,97]. However, in awake conditions, the LC cells display two different modes of firing patterns, including phasic and tonic firing. LC firing tonically at 1–3 Hz is related to the arousal, whereas phasic firing takes place in short bursts of 8–10 Hz in the presence of salient stimuli (for decision making and attention) [94,95,96,97,98]. The type of neuronal activity in the LC is important due to its role in eliciting a consistent behavioral response, maintaining the wakefulness and regulating the sleep and awake cycle, as well as the transition between both.

The majority of the LC (90%) is made of noradrenergic, tyrosine hydroxylase-positive neurons, and the rest of the region consists of non-noradrenergic neurons, such as serotonergic and GABAergic neurons [99,100]. Besides NA, there are a number of important peptides generated by the LC such as vasopressin, somatostatin, neuropeptide Y, enkephalin, neurotensin, corticotropin-releasing hormone and galanin, which contribute to the complexity of the LC system and the processes in its projection areas [101]. Studies also showed that noradrenergic neurons co-express galanin and neuropeptide Y [93,101]. Galanin attracts special attention due to the fact that 80% of the LC neurons co-express galanin and NA [102]. The net impact of this co-expression in LC target regions is still unknown. 

## 4. Noradrenaline

NA, which is also called norepinephrine, is a monoamine neurotransmitter (catecholamine) [103,104]. The initial step of the NA synthesis is conversion of the tyrosine through tyrosine hydroxylase with the formation of L-dihydroxyphenylalanine (L-DOPA) which forms dopamine by DOPA decarboxylase. After the vesicular monoamine transporter conveys dopamine into synaptic vesicles, it is converted to NA through the DBH [105]. The LC has a very broad projection system and noradrenergic fiber density can vary within cortical and subcortical structures. Generally, the strongest innervation was observed in cortical layer III and IV while layer I received fewer projections. This specificity is apparent in primates; however, it is less obvious or absent in rodents [106,107].

**Figure 5:**
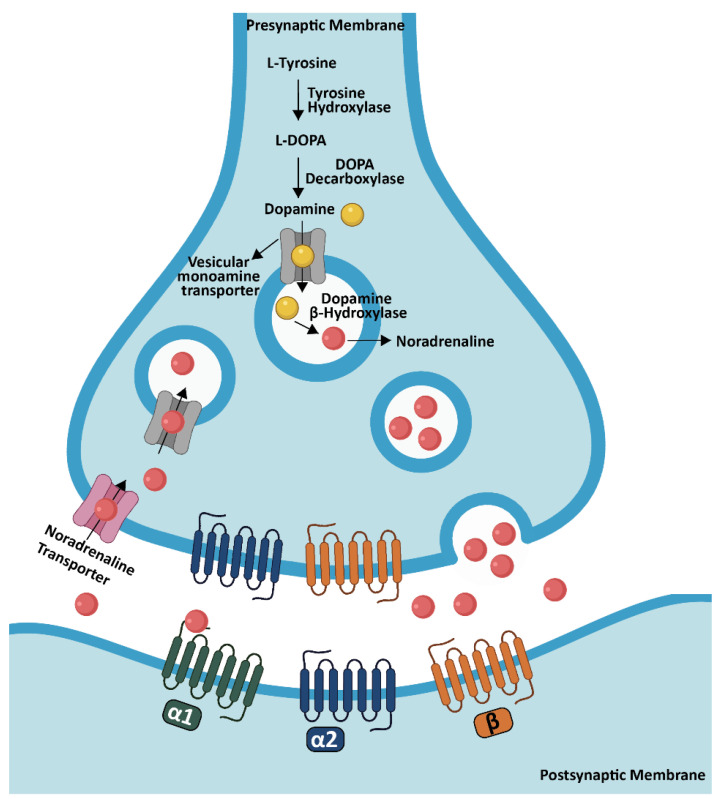
NA synthesis in the brain. NA synthesis is performed from dopamine through DBH in the LC and transported into synaptic vesicles by a vesicular monoamine transporter. After NA is released into the synaptic cleft, it binds to specific adrenergic receptors in order to activate the signaling for the specific task. Created partially with BioRender.com (accessed on 28 June 2021).

NA binds to G protein-coupled receptors which consist of three different types, including α1, α2, β, and each of them has several subtypes [108]. In the brain, α1 and β receptors are mainly present at postsynaptic sites where they perform excitatory actions, while α2 receptors are both at pre- and post-synaptic areas where they exert inhibitory effects [109]. α1-adrenergic receptors, which are coupled to Gq proteins, display an intermediate binding affinity (around 300 nM) to NA. In the cortex, three subtypes are present: α1A, B, D receptors; within these subtypes, α1D has the highest cortical expression in superficial layers [108,110]. α2-adrenergic receptors, which belong to the Gi protein-coupled receptor super family, show the highest binding affinity to NA (around 50 nM). They are composed of three receptor subtypes: α2A, B, C, and among them, α2A receptors show the highest expression in cortical superficial layers [108,111]. Lastly, the group of β-adrenergic receptors also consist of three subtypes (β1, 2, 3), which are coupled to the Gs family. They have the lowest binding affinity to NA (around 0.7–0.8 µM). In addition, β1 and β2 subtypes are the most abundant types in cortex, especially in layer IV [108,112,113] (Figure 5).

This greater diversity of adrenergic receptors with several subtypes gives rise to different modulation effects according to where they are expressed; downstream, they trigger distinct intracellular signaling pathways and finally initiate different physiological functions in specific regions of the brain. The release of NA illustrates this point clearly. While NA release is facilitated via β2 receptors which act exceptionally as presynaptic receptors [114], inhibition of the release is regulated by presynaptic α2 receptors [105,109].

## 5. The Locus Coeruleus–Noradrenaline System in Cognitive Processes

The LC–NA system innervates almost the entire CNS. In addition to its role in promoting wakefulness [115], the LC–NA system plays important roles in learning, memory, modulation of attentional networks and encoding of sensory information [116]. The function of NA in memory consolidation, which is a vital process in establishing long term memories, has been well studied [117]. In addition, noradrenergic modulation of the amygdala is very important for the formation of emotional memory. Interactions between stress hormones such as epinephrine, glucocorticoids and opioids, which are released in the basal lateral amygdala and the noradrenergic input from the LC in the same region, promote memory consolidation [118,119]. In mice, this can be seen in the experimental paradigms of foot shock stimulation or tail pinch responses. In these experiments, NA concentrations are increased in the rat amygdala [120,121]. The change in NA release within the amygdala might be critical for regulating memory consolidation and the degree of this change determines long-term memory for the specific experience [122]. Several animal experiments proved that during memory encoding or right after the behavioral training, injecting NA into the brain could improve memory performance [123,124], while blocking adrenergic receptors decreased memory functions [125]. In the prefrontal cortex (PFC), NA is involved in the modulation of working memory. An example of this is a study carried out on α-adrenergic receptors. The data proved that NA facilitates working memory through α2 receptors. In contrast, NA acting on α1 receptors inhibits working memory function [108].

Another significant aspect is the role of NA in LTP formation. Depletion in NA reduced the population spike rate in the dentate gyrus [126]. However, in contrast to previous findings, an increase is observed in the magnitude and duration of LTP in the CA3 subfield as a result of NA application [127]. It is important to mention that these effects are mediated by β-adrenergic receptors [128]. NA functioning via β-adrenergic receptors not only improves memory formation, but also possesses a greater impact on the induction of LTP [129]. However, with regard to long-term synaptic plasticity, α1- and β-adrenoreceptors have opposing effects. Studies proved that in the upper layers of the visual cortex, β-adrenoreceptors give rise to an enhancement of synaptic strength, whereas α1-adrenoreceptors create a decline in synaptic strength [130]. NA is also important for long term depression (LTD), which means a decrease in synaptic efficacy [131]. LTD takes place after the activation of N-methyl-D-aspartic acid (NMDA) receptors together with phospholipase C (PLC) by multiple neurotransmitter receptors coupled to Gq proteins. Recent data reported that NA can promote LTD induction of glutamate synaptic transmission in the cortex. Overall, these results showed that one of the important characteristics of the noradrenergic system is its bidirectional regulation of long term synaptic plasticity [132,133].

## 6. Importance of the Locus Coeruleus in Alzheimer’s Disease

During normal aging, the number of the LC cells and NA concentration in the brain declines, and by the age of 90 years, there is a loss of 25% of LC neurons and a 50% reduction in NA levels in the brain [134]. However, in AD, LC degeneration already occurs at early stages and is even detectable decades prior to visible neurocognitive signs [135,136,137]. There have been numerous studies which suggest a correlation of the LC degeneration with Aβ plaque load in the brain, NFTs and the severity of dementia [138]. In AD patients, up to 50% of the loss in the LC cells is found within the rostral nucleus and a 31% decrease in the NA concentration in the mid temporal cortex [139]. NFT accumulation in the LC appears in aged individuals, in patients with cognitive deficits [137] and in AD patients [140,141,142]. Previous studies have also shown that LC neuronal loss is related to increased cortical Aβ and NFT accumulations and it leads to a decrease in NA concentrations in cortex and hippocampus [138,140,141,142,143]. 

In addition to clinical studies, more specific studies aiming to unravel the underlying mechanisms were performed in rodent models (Table 1). In the light of the above mentioned studies on the LC–NA system, it now widely accepted that the LC degenerates in AD [141] and during aging [144], which subsequently leads to decreased levels of NA and its transporter [145] in AD patients [146]. The role of the NA transporter is the reuptake of synaptically released NA into the presynaptic neuron and thus restricts sustained effects of NA at the synapse [147,148]. It was previously shown that the levels of NA transporter were already decreased in the LC of APP/PS1 mice at the age of 6.5 months (age of plaque onset) compared to age-matched control animals. Likewise, cortical NA transporter innervations were also diminished in both 6.5 and 12.5 months old APP/PS1 animals compared with their wild type controls [149]. Taken together, the study indicated that AD pathology in murine models gives rise to a decline in NA transporter-immunoreactivity within the LC and its projection areas in cortex [149]. This decrease in NA transporter levels may result in elevated synaptic levels of NA and consequently reduced intraneuronal levels of NA [147]. 

N-(2-chloroethyl)-N-ethyl-bromo-benzylamine (DSP4) is a neurotoxin, which selectively damages LC neurons and fibers [150]. In APP23 mice, DSP4-induced LC degeneration not only increased Aβ deposits, but also resulted in neuronal loss in the hippocampus and frontal cortex, and worsened memory deficits [151]. In a different study, in order to investigate the consequences of NA loss in a mouse model of AD, APP/PS1 mice were crossbred with DBH^(−/−)^ animals specifically lacking NA, but exhibiting normal LC neuron numbers, LC fiber distribution and LC co-transmitter levels [88]. Findings showed that NA deficiency downregulated some of the learning-associated synaptic proteins, such as β-CaMKII in DBH^(−/−)^/APP/PS1 mice, and proved that a lack of NA impaired LTP [88]. Moreover, LC degeneration activates microglia and astrocytes, resulting in elevated levels of inducible nitric oxide synthase (iNOS) and NO-mediated peroxynitrite formation in both astrocytes and neurons [151]. Peroxynitrite is a free radical, which is highly toxic for the cells and it might contribute to the cell death, which is observed in the study [151]. Peroxynitrite also nitrates Aβ at the tyrosine residue (at position 10), thereby changing its propensity to aggregate [152]. NA affects AD pathology by reducing microglial activation [153], while ablation of NA inhibits the recruitment of microglia to Aβ plaques and leads to a decline in microglial phagocytosis [153]. Kummer et al. examined the consequences of early loss of LC neurons by crossing APP/PS1 mice with Ear2 deficient animals, which lack the orphan nuclear receptor that is necessary for the development of LC neurons [154]. These mice showed strong reductions of NA levels in all LC projections areas, including the olfactory bulb, frontal cortex, hippocampus, and cerebellum. Findings also pointed out that APP/PS1/Ear2^(−/−)^ mice showed impaired LTP together with a loss of spatial memory function [154]. It is also important to highlight the fact that analysis of postsynaptic proteins displayed alterations in NMDA receptor composition (reduced expression of NMDA2A subunit and increased levels of NMDA receptor 2B) which may be critical for receptor conductance and gating [154].

Similarly, in tau-based models, the ablation of the LC with the help of DSP4 treatment caused deteriorations in spatial learning and memory deficits [155]. Of note, in this model, LC deficits shortened the life span of the animals [155]. Taken together, these results provide insights into the role of the LC–NA system in AD pathology. According to the findings, the LC–NA system has a significant influence on learning, memory, synaptic integrity, and neuroinflammation, all of which are significantly associated with AD progression.

**Table 1:** In vivo models used to investigate the role of LC degeneration in AD progression. In order to examine the consequences of the LC degeneration in AD in general, either knockout mouse models, such as DBH^(−/−)^, Ear2^(−/−)^, or selective neurotoxins, such as DSP4, have been used in combination with amyloid or tau transgenic mice. DSP4 is a selective neurotoxins and it destroys the NA terminals originating from the LC [156]. DBH^(−/−)^ animals are lacking NA since birth [157]. Ear2^(−/−)^ mice lack ∼70% of the LC neurons projecting to the hippocampus and neocortex [158] from birth.

Animal Model	Animal Species	LC Degeneration Model	References
Aggregated Aβ 1–42 injection	Rat	DSP4	Heneka et al., 2002 [159]
Aggregated Aβ 1–42 and human recombinant IL-1β injection	Rat	DSP4	Heneka et al., 2003 [160]
APP23	Mouse	DSP4	Heneka et al., 2006 [151]
TASTPM	Mouse	DSP4	Pugh et al., 2007 [161]
H6	Mouse	DSP4	Kalinin et al., 2007 [162]
APP/PS1	Mouse	DSP4	Jardanhazi-Kurutz et al., 2010 [149]
APPV717I	Mouse	DSP4	Heneka et al., 2010 [153]
APP/PS1	Mouse	DSP4	Jardanhazi-Kurutz et al., 2011 [163]
APP/PS1	Mouse	DSP4	Rey et al., 2012 [164]
P301S	Mouse	DSP4	Chalermpalanupap et al., 2018 [155]
APP/PS1	Mouse	DBH^(−/−)^	Hammerschmidt et al., 2013 [88]
P301S	Mouse	DBH^(−/−)^	Kang et al., 2020 [165]
APP/PS1	Mouse	Ear2^(−/−)^	Kummer et al., 2014 [154]
APP/PS1	Mouse	-	Liu et al., 2013 [166]
5XFAD/BDNF KO * * Viral injection based local knocking out strategy for BDNF expression	Mouse	-	Braun et al., 2017 [167]
APP/PS1	Mouse	-	Cao et al., 2021 [168]

## 7. The Locus Coeruleus–Noradrenaline System and Microglia

The LC, which is also known as “blue spot” [105], is the major source of cerebral NA [169]. Next to its above described role as a classical neurotransmitter, NA has been identified in modulating not only the activity of neuronal cells but also non-neuronal cells, such as microglia and astrocytes [170,171,172]. 

Such modulating activity includes anti-inflammatory effects on microglial cells and the suppression of inflammatory markers, such as NOS-2, IL-1β, TNF-α and IL-6, by binding to β-adrenergic receptors and activation of cAMP signaling pathways [173,174,175]. Suppression of pro-inflammatory cytokine release may be crucial to protect neurons from adverse effects of neuroinflammation. Findings showed that NA protects cortical neurons against the detrimental effects of microglial activation and increases neuronal survival [176]. The critical role of NA on microglia is further supported via a study conducted by Heneka and colleagues detailing how NA influences microglia phenotypes in the presence of AD pathology [153]. The study revealed that NA treatment reduced the expression of pro-inflammatory gene expression, including TNF-α, CCL2 (MCP1), iNOS, and COX2, through microglia exposed to fibrillar Aβ1–42. Furthermore, it was shown that NA decreased the levels of TNF-α secretion and suppressed the production of CCL2, CCL3, CCL5 in Aβ-induced primary microglia cells [153]. The aforementioned paper also aimed at validating some important parameters in APP transgenic mice. In line with previous reports in vitro, NA ablation by DSP4 treatment in aged APP V717I transgenic mice enhanced inflammatory gene transcription and reduced microglial Aβ clearance by phagocytosis [153]. In addition, the study performed by Chalermpalanupap assessed the impact of LC degeneration in a murine model of tau-mediated neuropathology [155]. These experiments provided evidence for increased microglial activation and hippocampal tau burden in DSP-4 treated P301S tau-transgenic mice. 

Several studies have revealed that adrenergic receptors, specifically β-adrenergic signaling, play an important role in the context of neuroinflammation. This is exemplified in the work undertaken by Evans et al. [177]. Their findings showed that chronic metoprolol (β-adrenergic receptor antagonist) treatment enhanced inflammatory gene expression in the brains of female APP mice. In addition, conditional deletion of myeloid lineage-specific β1- or β2-adrenergic receptors in wild type mice provoked neuroinflammation in response to systemic LPS administration and increased levels of TNF-α, IL-1β and IL-6 [177]. Together, these results further highlight that NA not only represents a neurotransmitter, but a master regulator that controls inflammatory processes in the brain as well.

Using two-photon laser scanning in vivo microscopy of CX3CR1-EGFP mice, two more recent studies advanced our knowledge further by showing that awake and anesthetized conditions may influence microglial dynamics [178,179,180]. It is well-known that LC terminals release NA in order to promote wakefulness. The two studies illustrated, by using in vivo imaging, that NA reduces microglial arborization, surveillance, and injury response through the β2-adrenergic receptors. Additionally, NA also reduces the amount of time and contact areas that microglia spend with neuronal dendrites [178,179,180]. For instance, dexmedetomidine (DEX), which acts as a sedative by reducing NA release from the LC, enhanced microglial arborization and surveillance; however, optogenetic stimulation of the LC by channelrhodopsin led to reductions in process surveillance dynamics in DEX treated mice [179]. In addition to this, intracerebral NA administration to the mice under isoflurane prevented anesthesia-induced effects on microglia [178]. These findings were taken by examining further β2-adrenergic receptors agonist and antagonist treatments in mice. Clenbuterol, a β2-adrenergic receptor agonist, decreased microglial motility, arbor complexity and process coverage in fentanyl-anesthetized mice [179]. In line with this, ICI-118,551 (β2-adrenergic receptors antagonist) treatment of awake mice created an opposite effect, as expected, and enhanced microglial surveillance, motility and ramification [178,179]. These data further highlight the impact of NA and β2-adrenergic receptors on microglial dynamics and impressively display the power of intravital intracerebral microscopy.

## 8. Clinical Trials for Alzheimer’s Disease Based on the Locus Coeruleus–Noradrenaline System

AD causes an immense psychopathological and economic burden for patients and caregivers all over the world. Building upon the knowledge that the LC is one of the important brain regions which undergo early and detrimental changes in the course of AD, several laboratories performed extensive in vivo research and have recognized the fact that the LC–NA system may represent a potential target for disease-modifying therapies during AD progression. According to the efficacy check, noradrenergic drugs might offer a safe and effective treatment for AD. In a study, the outcomes of clinical trials from 1980 to December 2021 were analyzed using several databases including MEDLINE, Embase, and ClinicalTrials.gov. The results of a meta-analysis of 10 trials (consisting of 1300 patients) out of 19 trials (consisting of 1811 patients) showed a small but significant positive effect on global cognition in AD patients [181]. However, no significant effect was observed for attention improvement. Furthermore, a meta-analysis was performed on 8 clinical trials to study the effects of noradrenergic drugs on apathy (Apathy is a common change in behavior in AD) and the results indicated a large positive effect towards noradrenergic-based therapy [181]. So far, several compounds have been tested during clinical trials which specifically target the noradrenergic system.

Mirtazapine is an antagonist for 5-HT2A, 5-HT2C, 5-HT3, H1 and α2 receptors, and increases the release of both serotonin and NA [182]. It is prescribed for the treatment of depression symptoms. Depression is also prevalent among AD patients [183]. According to the literature, mirtazapine improved depression and anxiety, as well as insomnia and weight loss in AD patients [184]. An additional promising finding was observed in agitated patients with AD. In a pilot study, it was found that mirtazapine treatment showed beneficial effects and helped AD patients who were agitated, without causing significant adverse effects or cognitive impairment [185]. These results provide evidence for the beneficial effects of mirtazapine on depressed and agitated AD patients, possibly due to serotonergic and noradrenergic neurotransmission in modulating mood [184]. Despite the above findings, another clinical trial found negative results. Compared to placebo, mirtazapine and sertraline (serotonin reuptake inhibitor) were found to have no effect on AD patients diagnosed with clinically significant depression, a fact that is surprising given that these medications are the most commonly used antidepressants and prescribed for the treatment of AD depression [186]. In addition, a very recent clinical study of mirtazapine on agitated AD patients not only showed negative findings compared to placebo, but also findings leading to a potentially higher mortality rate [187]. Therefore, routine prescription of antidepressants should be reconsidered.

Monoamine oxidases (MAO-A and MAO-B) are mitochondrial-bound enzymes that are highly expressed in neuronal and gastrointestinal tissues in mammals. They catalyze the oxidative deamination of neurotransmitters, including NA, dopamine, tyramine, and serotonin. The oxidative deamination results in the formation of some neurotoxic products, including aldehyde, ammonia, and H_2_O_2_ [188]. The expression of MAO can be altered in AD patients. For instance, compared to healthy controls, mRNA levels of MAO-A and MAO-B were found to be significantly higher in AD brains [189]. Enhanced MAO activity results in decreased monoaminergic neurotransmission, which can lead to neurodegeneration [188,190]. The following study revealed that overexpression of MAO-B was observed in reactive astrocytes surrounding Aβ plaques [191]. As previously mentioned, enhanced levels of MAO-B can lead to an increased number of free radicals and H_2_O_2_. It is well-known that oxidative stress influences the formation of Aβ plaques, thus potentially accelerating the progression of AD [190]. MAO inhibitors may therefore play a role in neuroprotection and have therapeutic potential in the treatment of AD. Selegiline is one of the irreversible selective MAO-B inhibitors. The results of a controlled clinical trial examining the effects of selegiline on AD patients with moderate severity showed that, even though it did not improve cognitive test scores, it slowed disease progression [192]. In another clinical trial using lazabemide (a highly selective, reversible, inhibitor of MAO-B), less cognitive decline was observed in comparison to placebo. In addition, lazabemide was well tolerated for long-term treatments [193]. Likewise, in another study, it was demonstrated that the use of L-deprenyl, a selective MAO-B inhibitor, had positive effects on cognitive factors in AD patients, including memory and attention,. The drug is apparently well-tolerated as well [194].

An NA transporter takes NA up from the synaptic cleft into the presynaptic neuron to terminate noradrenergic neurotransmission [195]. By blocking the function of NA transporters but not the other monoamine transporters, NA reuptake inhibitors increase the concentration of extracellular NA in the periphery and brain, as well as the synaptic availability of NA [196,197,198]. Previously, atomoxetine, a clinically approved NA transporter inhibitor, was investigated for its efficacy in improving cognitive performance in patients with mild to moderate AD [199]. After a six-month treatment period, no significant improvements were noted in cognitive function, global clinical impression, or neuropsychiatric symptoms among AD patients who were also taking stable doses of cholinesterase inhibitors as combination therapy [199]. In contrast, a very recent clinical trial conducted by Levey et al. demonstrated that atomoxetine is beneficial for individuals living with mild cognitive impairment due to AD [200]. A small but significant reduction in both total tau and pTau181 levels in cerebrospinal fluid (CSF) was observed after six months of atomoxetine treatment, indicating a slowing of neurodegeneration. It could be related to the ability of NA to disrupt tau protofilaments and facilitate tau degradation. On the other hand, measurements of Aβ42 in the CSF showed no change. Since atomoxetine is a selective NA transporter inhibitor, the levels of NA and dopamine, the primary substrates of the NA transporter, were increased in CSF as expected. However, no clinically significant changes were observed in cognitive function as measured by the ADAS-Cog (AD Assessment Scale-Cognitive Subscale). Based on further analysis, several inflammatory analytes as well as synaptic and glial immunity biomarkers in CSF were found to be positively associated with treatment effects. Atomoxetine induced the amount of proteins in CSF that take part in synaptic function and metabolism, whose expression levels were decreased in brains with mild cognitive impairment. A possible explanation may be that atomoxetine has a tendency to normalize CSF protein levels to reverse the pathophysiology of the brain. In addition, atomoxetine treatment also helped to reduce the levels of the pro-inflammatory cytokines including CDCP1, CD244, and TWEAK in CSF. Overall, this clinical trial provided a promising outcome due to its potential disease-modifying capabilities [200].

Neuromodulation can be used to regulate cognition by stimulating a specific neuronal pathway. For instance, deep brain stimulation strategies might be helpful to decrease the rate of hippocampal atrophy as well as to enhance brain connectivity and memory [201]. Vagus nerve stimulation (VNS) plays an important role in this concept. The vagus nerve is the longest cranial nerve in the body, which is also called the “wanderer nerve”. It originates in the medulla oblongata and comprises motor and sensory functions on both the afferent and efferent regards [202,203,204]. VNS is used in the treatment of a wide range of neurological disorders, including epilepsy, depression, stroke, and tinnitus [205]. VNS affects the concentrations of serotonin, NA, and dopamine but in this part of the review, the main focus will be primarily on noradrenergic alterations. Key neural mechanism of VNS are as follows: The sensory vagal afferent cell bodies are located in the nodose ganglia and transfer the information to the nucleus tractus solitarii (NTS). NTS sends the vagal sensory information to different brain regions including the rostral ventrolateral medulla, amygdala, thalamus and the LC. Since the LC receives projections from NTS, which means that the LC receives afferent input indirectly from the vagus nerve, VNS initially leads to an increase in the firing activity of noradrenergic neurons and subsequently those of serotonergic neurons. As a result of the stimulation, NA concentration is enhanced in the brain [204,206,207,208,209]. Due to the known beneficial effect of NA on learning and memory, clinical trials were conducted to study the effects of VNS on human cognition. A clinical study performed on 45 ± 13 years old refractory epilepsy patients showed that VNS improved working memory performance and increased visual attention [210]. A recent clinical research by Jacobs et al. investigated the effects of transcutaneous VNS (tVNS), a non-invasive alternative on memory improvement on elderly individuals (mean age 60.57 years) [211]. Results indicated that participants performed better on the memory task during tVNS. Considering the fact that the LC is affected by tau burden early in life, the results obtained from this study are of particular importance. Based on the results of the study, it is evident that alterations in the LC due to tVNS are capable of improving memory performance in older individuals [211]. In a pilot study among AD patients, VNS showed a positive effect on cognition [212]. The cognitive function of patients was already improved 3 months after VNS treatment based on ADAS-cog and MMSE (Mini-Mental State Examination) scores. These improvements were further enhanced at 6 months of treatment, indicating that VNS treatment has a beneficial effect on cognitive performance. Moreover, it is important to emphasize that VNS is well tolerated by patients with only mild and transient side effects [212]. According to the follow-up results of this study after one year of treatment, an improvement in cognition is still observed using the MMSE score [213]. In the course of the one-year study period, no significant decrease in mood or behavior was observed. Further experiments were carried out to determine tau levels in CSF. According to the results, CSF tau showed a median drop of 4.8%, whereas phosphotau showed a 5.0% increase. The reason for this could be that VNS may contribute to ameliorating axonal degeneration, but does not affect the phosphorylative processes in AD. As a final note, VNS was well tolerated by AD patients over a period of one year [213]. 

Additionally, there are further ongoing clinical studies: One of them is testing a noradrenergic add-on therapy with guanfacine (NorAD) [214,215]. An α2A adrenergic receptor agonist, guanfacine, is used in the treatment of attention deficit hyperactivity disorder and has been shown to improve cognitive functions including working memory and attention [216]. The purpose of NorAD is to evaluate the efficacy of guanfacine in combination with cholinesterase inhibitors on AD patients, as well as compare it to placebo [214,215]. The study started in January 2019 and was funded by the UK National Institute of Health Research [214,215]. Another study investigates the effect of selective VNS on mild-to-moderate AD patients to regulate the activity of the LC–NA network [217]. After the stimulation, the impact on cognitive and memory function will be analyzed. This clinical study will be conducted at the Xuanwu Hospital of Capital Medical University [217]. There are other trials being conducted examining the LC–NA system in healthy elderly individuals. As an example, one clinical trial examines the effects of tVNS on different brain regions related to memory and attention in healthy human volunteers between the ages of 60 to 80 [218]. This study started in January 2021 at the Maastricht University Medical Center in collaboration with the University of Liege [218]. Another clinical study examines the effect of tVNS on cognition in older adults [219]. This study started in November 2021 and is being conducted at the Massachusetts General Hospital [219].

## 9. Conclusions and Future Directions

Over the past decades, basic and clinical research have contributed to the understanding of the biological mechanisms underlying AD pathogenesis. Nevertheless, efforts to develop appropriate therapies for the prevention or slowing of AD are still not as effective as they should be. The perspective of Thomas Edison (“I have not failed. I have simply identified 10,000 ways that will not work”) can, however, serve as a driving force in this concept, since each failed trial contributes to the identification of a potential therapy by excluding the unsuccessful ones, and can also serve as a useful tool in adjusting the new treatment courses for future.

On the basis of our knowledge, it is evident that the LC–NA system is a key neuroregulator for cognitive functions, such as attention, learning, and memory, as well as immune responses. This picture has been elaborated using advanced methodological techniques which provide a great opportunity for precise investigation and modulation of LC–NA system. Due to the fact that the LC is the region of the brain where initial pathophysiological AD processes start, it can be used as an early diagnostic marker and routinely checked in hospitals. 

For AD patients, the LC–NA system represents a promising target for the development of novel therapies that can either improve cognition or modify disease progression. To determine the optimal amount of NA required to reduce or interfere with the pathological sequelae of AD, it is crucial to know the concentration of NA in age-matched healthy individuals. A first critical step in this direction is to measure NA levels in the brains of healthy subjects and AD patients in large longitudinal studies. Unfortunately, the amount of actual data and ongoing clinical research on this subject is still limited. Alternatively, checking the activity of LC neurons would be another option. Since this cannot be studied non-invasively in the human brain, alternative techniques such as pupil dilation and the p300 event-related potential could be good options to measure LC activity. The future development of high precision treatments requires well-defined and validated measurements to characterize the alterations and potential treatment responses in the LC–NA system during aging and AD.

All of these studies have the ultimate goal of reversing the pathology or slowing down the detrimental effects of AD pathogenesis. There have been many studies investigating different noradrenergic molecules (e.g., several agonists, antagonists, reuptake inhibitors, etc.); however, combination therapy approaches may provide better results. Therefore, targeting more than one pathway might be more effective to treat a multifactorial neurodegenerative process. Last but not least, living a healthy lifestyle (diet, sleep, exercise, etc.) can also help prevent or delay AD symptoms, as well as extend life expectancy.

## Data Availability

Not applicable.
